# Results of Treatment with Modern Fractionated Radiotherapy, Contemporary Stereotactic Radiosurgery, and Transsphenoidal Surgery in Nonfunctioning Pituitary Macroadenoma

**DOI:** 10.3390/jcm8040518

**Published:** 2019-04-16

**Authors:** Ping-Kun Hsiao, Chia-Lun Chang, Kevin Sheng-Po Yuan, Alexander T.H. Wu, Szu-Yuan Wu

**Affiliations:** 1 Department of General Surgery, Wan Fang Hospital, Taipei Medical University, Taipei 11031, Taiwan; 86301@w.tmu.edu.tw; 2 Department of Internal Medicine, School of Medicine, College of Medicine, Taipei Medical University, Taipei 11031, Taiwan; richardch9@hotmail.com; 3 Department of Hemato-Oncology, Wan Fang Hospital, Taipei Medical University, Taipei 11031, Taiwan; 4 Department of Otorhinolaryngology, Wan Fang Hospital, Taipei Medical University, Taipei 11031, Taiwan; dryuank@gmail.com; 5 Ph.D. Program for Translational Medicine, Taipei Medical University, Taipei 11031, Taiwan; chaw1211@tmu.edu.tw; 6 Department of Radiation Oncology, Wan Fang Hospital, Taipei Medical University, Taipei 11031, Taiwan; 7 Department of Radiology, School of Medicine, College of Medicine, Taipei Medical University, Taipei 11031, Taiwan

**Keywords:** nonfunctioning pituitary macroadenoma, transsphenoidal surgery, stereotactic radiosurgery, fractionated radiotherapy, recurrence, mortality

## Abstract

Background: To compare the effects of contemporary stereotactic radiosurgery (SRS), modern fractionated radiotherapy (FRT), and transsphenoidal surgery on nonfunctioning pituitary macroadenoma. Methods: We enrolled patients with nonfunctioning pituitary macroadenoma. To compare treatment outcomes, the patients were categorized into three groups according to the treatment modality: group 1, patients receiving modern FRT; group 2, patients receiving contemporary SRS; and group 3, patients receiving transsphenoidal surgery. Results: In total, 548 patients with nonfunctioning pituitary macroadenoma were selected for our study. Univariate and multivariate Cox regression analysis results indicated that the treatment modalities were significant independent prognostic factors. In multivariable Cox proportional hazard regression analysis, the adjusted hazard ratios (aHR; 95% confidence interval (CI)) of local recurrence were 0.27 (0.10–0.91) and 1.95 (1.25–2.37) for the SRS and transsphenoidal surgery cohorts, respectively, in comparison with the FRT cohort. The aHR (95% CI) of all-cause mortality was 1.03 (0.68–1.56) for the transsphenoidal surgery cohort in comparison with the FRT cohort, without statistical significance. However, the aHR (95% CI) of all-cause mortality was 0.36 (0.15–0.85) for the SRS cohort in comparison with the FRT cohort. Conclusion: Contemporary SRS has optimal effects on local recurrence and survival compared with modern FRT and transsphenoidal surgery. Modern FRT is associated with more favorable local control and equal survival compared with transsphenoidal surgery.

## 1. Introduction

Most patients with pituitary adenoma present with signs and symptoms of hormone hypersecretion [1]. However, 25% to 35% of pituitary adenomas are clinically nonfunctioning or “silent” [1,2]. Patients with clinically nonfunctioning adenoma most often present with neurologic symptoms [1,2,3]. Clinically nonfunctioning adenoma is difficult to identify because its secretory products usually do not cause a recognizable clinical syndrome and because the products are often secreted inefficiently; thus, their serum concentrations and subunits are often minimally abnormal or not abnormal at all [1,2,3]. Consequently, the adenomas are typically not detected until they become sufficiently large to cause neurologic symptoms, which most often include impaired vision due to pressure on the optic chiasm [4].

Nonfunctioning pituitary macroadenoma is typically diagnosed on the basis of headaches or visual loss [1]. Once identified, transsphenoidal surgery should be promptly planned for those with impaired vision and should be considered for those at a high risk of loss of vision [4,5]. The risk of surgical complications, including cerebrospinal fluid leakage, fistula, meningitis, and visual field defects, is inversely proportional to the experience of the surgeon performing the transsphenoidal surgery [4,6].

Radiotherapy (RT) is also considered for clinically nonfunctioning pituitary macroadenoma [5]. In this setting, the goal of RT is to stop nonfunctioning pituitary macroadenoma growth [5]. Shrinkage, partial or complete, may occur [5,7,8]. Current modern RT employs more sophisticated imaging and targeting techniques than those previously available; however, most published papers are based on older imaging techniques [5,7,8]. Newer techniques such as intensity-modulated radiation therapy (IMRT) or volumetric arc therapy (VMAT) reduce the incidence and severity of the side effects [9,10]. Fractionated RT (FRT) is the most common method by which RT is delivered for most indications of radiation treatment [11]. In stereotactic radiosurgery (SRS), a single high dose of radiation therapy is delivered using a high-precision localization system to treat a small target [12]. It is the most effective and safe technique when treating small lesions and when an extremely accurate area can be targeted, allowing safety margins to be minimized and the total target size to be as small as possible [12]. With the progression of SRS techniques, the modern Linac-based radiosurgical systems (CyberKnife® system from Accuray, Sunnyvale, CA, USA and Novalis ExacTrac® X-Ray 6D systems, Munich, Germany) now regularly employ online cone beam computed tomography (CT) scanning for precision localization, and these systems irradiate both small and large complex-shaped lesions [13]. Many of these advances in SRS techniques include planning systems that enhance the conformity of dose distribution, as well as delivery systems that can more safely and efficiently deliver radiation doses in complex treatment plans [13]. No randomized trial has directly compared the aforementioned treatment modalities, and no study has evaluated patients with nonfunctioning pituitary macroadenoma receiving modern SRS, modern FRT, or transsphenoidal surgery. Optimal treatment paradigms are subject to institutional bias or timing of diagnosis. We conducted a nationwide population-based cohort study to investigate the effectiveness of modern SRS, modern FRT, and transsphenoidal surgery in patients with nonfunctioning pituitary macroadenoma.

## 2. Patients and Methods

We conducted a population-based cohort study using the Taiwan National Health Insurance (NHI) Research Database (NHIRD) linked to the Taiwan Cancer Registry Database (TCRD). The TCRD was established in 1979 and contains information of 97% of cancer cases in Taiwan [14]. The NHIRD includes all medical claims data on disease diagnoses, procedures, drug prescriptions, demographics, and enrollment profiles of all beneficiaries [15]. The NHIRD and TCR are linked by encrypted patient identifiers. NHIRD data are additionally linked to the Mortality Registry to ascertain the vital status and the cause of mortality of each patient. Using the data from the two databases, we selected patients diagnosed with nonfunctioning pituitary macroadenoma from 1 January 2006 to 31 December 2015. The follow-up period was from the index date to 31 December 2015. The index date was the date of RT in the FRT and SRS cohorts or the date of transsphenoidal surgery in the surgery cohort. Patients who received treatment more than three months after pituitary adenoma diagnosis were excluded from the study. Our protocols were reviewed and approved by the Institutional Review Board of Taipei Medical University (TMU-JIRB 201712019). The TCRD of the Collaboration Center of Health Information Application contains detailed cancer-related information on clinical stages, RT doses, and RT techniques [16,17,18,19,20,21,22]. The diagnoses of selected patients were confirmed on the basis of the two databases, and it was confirmed that patients newly diagnosed with nonfunctioning pituitary macroadenoma had no other cancers. The inclusion criteria were as follows: a diagnosis of nonfunctioning pituitary macroadenoma as well as a minimum adequate RT dose ≥45 Gy in the FRT cohort and a minimum adequate SRS dose ≥14 Gy in one fraction in the SRS cohort. Adjuvant RT, including SRS or FRT, was permitted in the surgery cohort. The exclusion criteria were as follows: a history of cancer before nonfunctioning pituitary macroadenoma diagnosis, missing sex data, unclear microadenoma or macroadenoma, and functioning pituitary adenoma with signs and symptoms of hormone hypersecretion. In addition, we excluded patients who underwent therapy for more than 12 weeks after nonfunctioning pituitary macroadenoma diagnosis and who did not receive modern RT techniques such as IMRT or VMAT in the FRT cohort or modern Linac-based radiosurgical systems (CyberKnife or Novalis ExacTrac X-Ray 6D systems) in the SRS cohort. Finally, we selected patients with nonfunctioning pituitary macroadenoma, and to compare their outcomes, they were categorized into the following groups on the basis of the treatment modality: group 1, patients receiving modern FRT; group 2, patients receiving modern SRS; group 3, patients receiving transsphenoidal surgery. The median total dose and fraction size for RT were 50.4 and 1.8 Gy, respectively, in group 1, and one dose of 18 Gy was administered in group 2.

Comorbidities were scored using the Charlson comorbidity index (CCI) [23,24]. Only comorbidities observed six months before the index date were included; comorbidities were identified according to the main International Classification of Diseases, Ninth Revision, Clinical Modification (ICD-9-CM) diagnosis codes for the first admission or more than two repeated main diagnosis codes for visits to the outpatient department. To ensure that the two RT cohorts did not include patients with poor performance related to their inoperable status, we also evaluated patients with an American Society of Anesthesiologists (ASA) physical status score, which indicated the balance between the number of healthy performance status indicators and tolerance to surgery in the two RT cohorts (Table 1).

After adjustment for confounders, the time-dependent Cox proportional hazard model was used to model the time from the index date to all-cause mortality or local recurrence (LR) in patients undergoing the treatments. The recurrence was defined as re-surgery, re-RT, or pharmacologic treatment after six months of the index date. Multivariable Cox regression analysis was performed to calculate the hazard ratio (HR) to determine whether factors such as different therapies, age, sex, CCI scores, ASA scores, residential area, and income level were significant independent predictors. The independent predictors were controlled for in the analysis, and the endpoints were the mortality rate and LR rate in the treatment cohorts, with group 1 (FRT) serving as the control arm.

The cumulative mortality or LR rates were estimated using the Kaplan–Meier method. Differences between the three treatment cohorts were determined using the log-rank test. After adjustment for confounders, all-cause mortality and LR rates were estimated using the time-dependent Cox proportional hazard model curves for overall survival (OS) or LR in patients undergoing different treatments. In multivariable analysis, HRs were adjusted for age, sex, CCI scores, ASA scores, residential area, income level, and different treatments. All analyses were performed using SAS (version 9.3; SAS, Cary, NC, USA). A two-tailed *p* < 0.05 was considered statistically significant.

## 3. Results

From 2006 to 2015, 548 patients with nonfunctioning pituitary macroadenoma were selected in our study (Table 1). Overall, 133 patients received modern FRT, 53 patients received modern SRS, and 362 patients received transsphenoidal surgery. No statistically significant difference was observed in sex, ASA scores, residential area, and income level between the three cohorts. However, compared with the transsphenoidal surgery cohort, the FRT and SRS cohorts exhibited higher CCI scores and included more older patients. The mean age of patients in the modern FRT, modern SRS, and transsphenoidal surgery cohorts was 52.7, 43.2, and 40.0 years, respectively, and the median follow-up duration was 4.1, 3.4, and 3.2 years, respectively. Age distribution by 10-year intervals was balanced among the three cohorts (Table 1). The crude LR rates were 14.3%, 5.7%, and 37.3% in the FRT, SRS, and transsphenoidal surgery cohorts, respectively. The crude overall mortality rates were 29.3%, 15.1%, and 24.6% in the FRT, SRS, and transsphenoidal surgery cohorts, respectively (Table 1).

According to the multivariable Cox proportional hazard regression analysis of the risk of LR among patients with nonfunctioning pituitary macroadenoma receiving different therapies, various treatments were significant independent prognostic factors for LR (Table 2). In multivariable Cox proportional hazard regression analysis, the adjusted HRs (aHRs; 95% confidence intervals (CIs)) of LR were 0.27 (0.10–0.91) and 1.95 (1.25–2.37) for the SRS and transsphenoidal surgery cohorts in comparison with the FRT cohort, respectively. Both univariable and multivariable Cox proportional hazard regression analyses indicated that SRS was associated with the lowest LR risk compared with the other treatments. The transsphenoidal surgery cohort showed a higher LR risk than the FRT cohort. In multivariable Cox proportional hazard regression analysis, the aHR (95% CI) of all-cause mortality was 1.03 (0.68–1.56) for the transsphenoidal surgery cohort in comparison with the FRT cohort, without statistical significance (Table 3). However, in multivariable Cox regression analyses, the aHR (95% CI) for significant independent prognostic factors for higher OS was 0.36 (0.15–0.85) for the SRS cohort in comparison with the FRT cohort. In addition, multivariable Cox regression analysis showed that older age and higher CCI scores were associated with poor OS (Table 3). The aHRs of all-cause mortality were 2.03 (1.16–4.31), 2.12 (1.52–6.56), 2.77 (0.83–3.77), and 2.99 (1.41–5.38) for the age groups of 40–49, 50–59, 60–69, and ≥70 years in comparison with the age group of 1 to 17 years. The aHRs (95% CIs) of all-cause mortality were 2.08 (1.33–3.26) and 4.56 (2.51–7.28) for CCI scores of 1–2 and ≥3, respectively, in comparison with the CCI score of 0 (Table 3).

**Figure 1:**
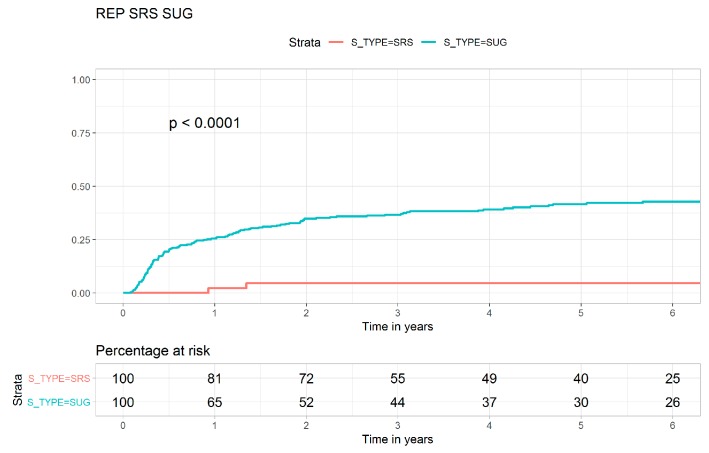
Kaplan–Meier curves for local recurrence in patients with nonfunctioning pituitary macroadenoma who underwent stereotactic radiosurgery or transsphenoidal surgery.

**Figure 2:**
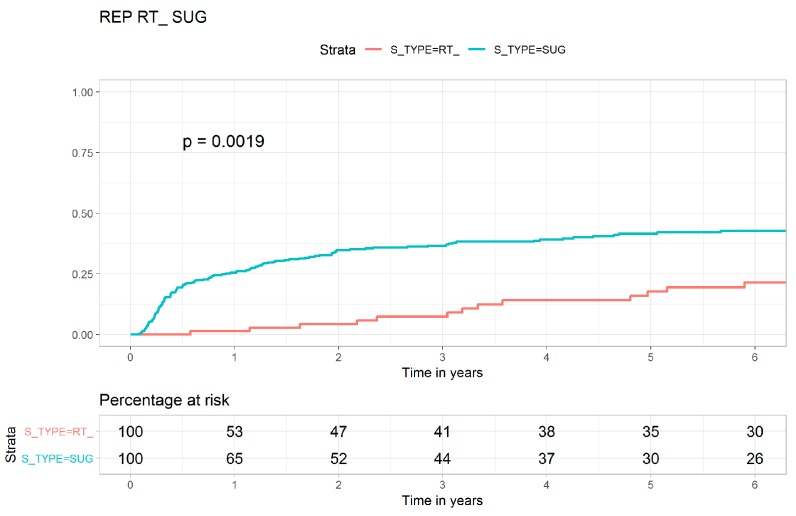
Kaplan–Meier curves for local recurrence in patients with nonfunctioning pituitary macroadenoma who underwent fractionated radiotherapy or transsphenoidal surgery.

**Figure 3:**
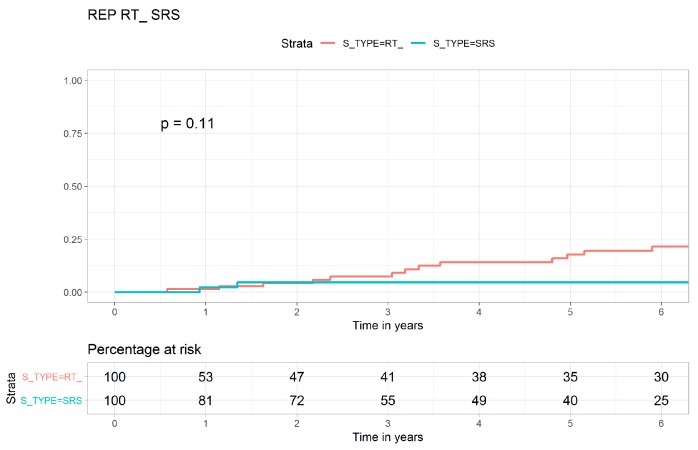
Kaplan–Meier curves for local recurrence in patients with nonfunctioning pituitary macroadenoma who underwent fractionated radiotherapy or stereotactic radiosurgery.

Figure 1, Figure 2 and Figure 3 show the Kaplan–Meier curves for the cumulative LR of the FRT, SRS, and transsphenoidal surgery cohorts. The LR risk was significantly higher in the transsphenoidal surgery cohort than in the SRS cohort (log-rank test, *p* < 0.0001, Figure 1). The LR risk was also significantly higher in the transsphenoidal surgery cohort than in the FRT cohort (log-rank test, *p* = 0.0019, Figure 2). The crude Kaplan–Meier curves for LR were not statistically different between the SRS and the FRT cohorts (log-rank test, *p* = 0.110, Figure 3). Supplemental Figures S1–S3 present the Kaplan–Meier curves for all-cause mortality in the FRT, SRS, and transsphenoidal surgery cohorts, and no statistically significant differences were observed between the three cohorts. The toxicities in patients with nonfunctioning pituitary macroadenoma who received FRT, SRS, or transsphenoidal surgery are shown in Table 4. There were no statistical differences in secondary primary brain or head and neck cancers, hypopituitarism, or visual field deficit between FRT, SRS, or transsphenoidal surgery.

## 4. Discussion

Pituitary adenoma is rare, has a variable presentation, and is often diagnosed on the basis of autopsy or image series [25,26,27,28,29]. The true prevalence of pituitary adenoma is likely to be underestimated because many nonfunctioning pituitary adenomas remain undiagnosed until they are very large or are identified incidentally in an imaging study conducted for unrelated reasons [26,27,28,29]. Gonadotroph adenoma is the most common pituitary macroadenoma, comprising approximately 80% of clinically nonfunctioning pituitary macroadenoma cases [30]. Other nonfunctioning macroadenomas are typically diagnosed when they become large enough to cause neurologic symptoms or a hormonal deficiency state and when an imaging study is performed for unrelated reasons [30]. Therefore, no standard treatment has been established for nonfunctioning pituitary macroadenoma. Surgery is the most common therapy for nonfunctioning pituitary macroadenoma [31]. However, the modern techniques of RT or SRS have improved the planning target volume coverage, have enabled sparing of critical organs and highly conformal dose distribution, and have facilitated the irradiation of both small and large complex-shaped lesions, while minimizing the dose to adjacent radiosensitive tissues [9,10,13]. The following research question arises: Is transsphenoidal surgery still the gold standard for symptomatic nonfunctioning pituitary macroadenoma in the era of modern RT? The present study is the first to evaluate the outcomes of the modern RT techniques of IMRT and VMAT in the FRT cohort and of the modern Linac-based radiosurgical systems (CyberKnife and Novalis ExacTrac X-Ray 6D systems) in the SRS cohort.

The goals of treatment in patients with nonfunctioning pituitary macroadenoma include the removal of pituitary macroadenoma as completely as possible to avoid recurrence [31]. However, in two case series, patients with nonfunctioning pituitary adenoma who underwent transsphenoidal surgery had higher recurrence rates of 19% and 34% [32,33]. In addition, patients with nonfunctioning pituitary macroadenoma treated with transsphenoidal surgery have impaired quality of life [34]. These findings show that transsphenoidal surgery is not the optimal procedure for nonfunctioning pituitary macroadenoma. However, no study has investigated whether modern FRT or contemporary SRS is more effective than transsphenoidal surgery for nonfunctioning pituitary macroadenoma. Our study is the first to show that contemporary SRS achieves optimal effects on local control and all-cause mortality in patients with nonfunctioning pituitary macroadenoma. In multivariate analysis, the SRS cohort had optimal local control and the highest overall survival compared with the transsphenoidal surgery and modern FRT cohorts (Table 2 and Table 3 and Figure 1). In addition, FRT with IMRT or VMAT also resulted in more favorable local control than transsphenoidal surgery (Table 2 and Figure 2).

As shown in Table 1, more older patients and patients with a higher CCI score were included in the SRS and FRT cohorts. Higher CCI scores and older age were poor prognostic factors for OS among patients with nonfunctioning pituitary macroadenoma (Table 3). However, even though patients with higher CCI scores and more older patients were included in the SRS cohort, the mortality risk was still significantly lower in the SRS cohort than in the transsphenoidal surgery cohort. Despite the competition of unclear bias to the endpoint of OS, the Cox model would be biased toward null with regard to the effect of SRS users having greater than expected proportions of underlying diseases and older age; hence, the conclusions of the current study should remain valid. This is the first study to demonstrate that SRS is more beneficial for OS than transsphenoidal surgery in patients with nonfunctioning pituitary macroadenoma.

As shown in Table 1, crude LR was the highest in the transsphenoidal surgery cohort compared with the SRS and FRT cohorts, which showed the least LR risk and intermediate LR risk in multivariate analysis (Table 2). Age, sex, CCI scores, ASA scores, income level, and residential area were not risk factors for LR. The skills of contemporary SRS have improved a lot in relative larger tumor volume, with the dose decreasing rapidly with the distance and lower irradiation dose to other brain tissue [13]. The learning curve of SRS might be also short, and SRS might require less hospital and surgeon experience [4] than transsphenoidal surgery [6]. Our findings are similar to those of a study that found more favorable brain local control in SRS than in surgery [35]. In this study, the crude Kaplan–Meier curves for LR were not statistically different between contemporary SRS and modern FRT cohorts (log-rank test, *p* = 0.110, Figure 3). In multivariate analysis, the SRS cohort showed more favorable LC than the modern FRT cohort; this may be because SRS and FRT might have different radiobiology effects on pituitary adenoma [36,37,38,39]. The growth of pituitary adenoma is slow, and a larger fraction size of irradiation may result in a higher local control rate [36,38,39,40,41]. In our study, most patients with nonfunctioning pituitary macroadenoma who underwent subtotal resection of an adenoma with suprasellar extension were at a high risk of recurrence. This finding is compatible with those of previous studies [33,42]. In addition, the differences in the indications for each modality are chosen according to the locations and sizes of nonfunctioning pituitary macroadenoma. In general, SRS is chosen for nonfunctioning pituitary macroadenoma that may be not close to the optic pathway and could be smaller than 3 cm in diameter, whereas transsphenoidal surgery or FRT is chosen for nonfunctioning pituitary macroadenoma that might be larger than 3 cm and closer to the optic pathway [43,44]. In the present study, Figure 1, Figure 2 and Figure 3 indicate SRS might be the optimal treatment for selected nonfunctioning pituitary macroadenoma.

The strength of our study is that it is the first to compare modern SRS, modern FRT, and transsphenoidal surgery to identify the optimal therapy for nonfunctioning pituitary macroadenoma. Furthermore, compared with previous studies, the current study had the largest sample size and the highest curative therapeutic consistency in modern RT techniques for nonfunctioning pituitary macroadenoma. Compared with modern FRT and transsphenoidal surgery, modern SRS for the treatment of nonfunctioning pituitary macroadenoma resulted in not only optimal LC but also optimal OS. The effect of modern FRT on LC was superior to that of surgery, and FRT and surgery showed comparable effects on OS. These findings should be considered in clinical practice and should be confirmed in future randomized controlled studies.

This study has some limitations. First, the toxicity of different treatments could not be determined; therefore, treatment-related mortality or morbidity estimates may have been biased. However, more older patients and patients with higher CCI scores were included in the SRS cohort than in the transsphenoidal surgery cohort. In the current study, the improved survival rate engendered by SRS may have been underestimated. Second, because all patients with nonfunctioning pituitary macroadenoma were enrolled from an Asian population, the corresponding ethnic susceptibility remains unclear; hence, our results should be cautiously extrapolated to non-Asian populations. Third, the diagnoses of all comorbidities were based on ICD-9-CM codes. Nevertheless, the National Taiwan Insurance Administration randomly reviews medical charts and interviews patients to verify the accuracy of the diagnoses, and hospitals with outlier chargers or practices may be audited and subsequently heavily penalized if malpractice or discrepancies are identified. Fourth, to prevent the creation of several subgroups, during analyses, the study patients were not separately categorized according to various adjuvant treatments administered after transsphenoidal surgery. Thus, the effects of different adjuvant treatments remain unclear. Therefore, although adjuvant SRS or FRT was administered after transsphenoidal surgery in our study, LC and OS were still poor in the transsphenoidal surgery cohort. The conclusions may not change if additional subgroups are created according to different adjuvant treatments. Fifth, since initial therapeutic decision depended on tumor size or symptoms, objective neurological deficits might be due to the selection bias in the retrospective cohort study. The tumor size or symptoms and objective neurological deficits were not recorded in the TCRD. Owing to the fact that nonfunctioning pituitary macroadenoma are rare, head-to-head randomized controlled trials might be completed difficultly. Large randomized controlled trials with adequate sample sizes have yet to compare the effects of contemporary SRS, modern FRT, and transsphenoidal surgery on nonfunctioning pituitary macroadenoma. This study is the first and largest to demonstrate that contemporary SRS has optimal effects on local control and survival compared with modern FRT at least. Accordingly, to obtain crucial information on population specificity and disease occurrence, a large-scale randomized trial comparing carefully selected patients undergoing suitable treatments is essential. Finally, the TCRD does not contain information on dietary habits, socioeconomic status, or body mass index, all of which may be risk factors for mortality. However, considering the magnitude and statistical significance of the observed effects in this study, these limitations are unlikely to affect the conclusions.

## 5. Conclusions

Contemporary SRS has optimal effects on LC and OS compared with modern FRT and transsphenoidal surgery. Modern FRT is associated with more favorable LC and equal OS compared with transsphenoidal surgery.

## 6. Novelty & Effect Statements

Large randomized controlled trials with adequate sample sizes have yet to compare the effects of contemporary stereotactic radiosurgery (SRS), modern fractionated radiotherapy (FRT), and transsphenoidal surgery on nonfunctioning pituitary macroadenoma. This study is the first to demonstrate that contemporary SRS has optimal effects on local control and survival compared with modern FRT and transsphenoidal surgery. Modern FRT is associated with more favorable local control and equal survival compared with transsphenoidal surgery.

## Figures and Tables

**Table 1:** Characteristics of patients with nonfunctioning pituitary macroadenoma who received fractionated radiotherapy, stereotactic radiosurgery, or transsphenoidal surgery.

	Fractionated Radiotherapy	Stereotactic Radiosurgery	Transsphenoidal Surgery	*p*-Value
	N, %	N, %	N, %	
Sex	133	53	362	0.877
Male	79 (59.4)	32 (60.4)	208 (57.5)	
Female	54 (40.6)	21 (39.6)	154 (42.5)	
Age				0.024
1–17	22 (16.5)	9 (17.0)	96 (26.5)	
18–29	11 (8.3)	7 (13.2)	48 (13.3)	
30–39	26 (19.5)	12 (22.6)	46 (12.7)	
40–49	24 (18.0)	8 (15.1)	67 (18.5)	
50–59	17 (12.8)	6 (11.3)	50 (13.8)	
60–69	19 (14.3)	3 (5.7)	35 (9.7)	
≥70	14 (10.5)	8 (15.1)	20 (5.5)	
Urbanization level				0.111
1 (most urbanized)	30 (22.6)	19 (35.8)	84 (23.2)	
2	17 (12.8)	6 (11.3)	69 (19.1)	
3	6 (4.5)	3 (5.7)	32 (8.8)	
4	13 (9.8)	6 (11.3)	27 (7.5)	
5 (least urbanized)	67 (50.4)	19 (35.8)	150 (41.4)	
Monthly income				0.583
≤NTD15,840	23 (17.3)	10 (18.9)	84 (23.2)	
NTD15,841–25,000	59 (44.4)	26 (49.1)	153 (42.3)	
≥NTD25,001	51 (38.3)	17 (32.1)	125 (34.5)	
CCI				0.029
0	93 (69.9)	38 (71.7)	286 (79.0)	
1–2	34 (25.6)	9 (17.0)	51 (14.1)	
3+	6 (4.5)	6 (11.3)	25 (6.9)	
ASA Scores				0.868
ASA = 1	84 (63.2)	32 (60.4)	232 (64.1)	
ASA > 1	49 (36.8)	21 (39.6)	130 (35.9)	
Local recurrence	19 (14.3)	3 (5.7)	135 (37.3)	<0.001
Radiation dose (median, Gy)	50.4	18	0	<0.001
Death				0.126
No	94 (70.7)	45 (84.9)	273 (75.4)	
Yes	39 (29.3)	8 (15.1)	89 (24.6)	

NTD, New Taiwan Dollar; Gy, gray; ASA, American Society of Anesthesiologists; CCI, Charlson comorbidity index.

**Table 2:** Cox proportional hazard regression analysis of the risk of local recurrence among patients with nonfunctioning pituitary macroadenoma receiving different therapies.

	Crude HR (95% CI)	Adjusted HR * (95% CI)	*p*-Value
Therapeutic modality (REF: Fractionated radiotherapy)			
Stereotactic radiosurgery	0.28 (0.10–0.95)	0.27 (0.10–0.91)	0.0345
Transsphenoidal surgery	2.12 (1.31–2.42)	1.95 (1.25–2.37)	0.0044
Sex (REF: male)			
Female	0.935 (0.73–1.23)	0.990 (0.77–1.12)	0.7397
Age (REF: 1–17)			
18–29	0.667 (0.4–1.12)	0.576 (0.34–1.98)	0.5120
30–39	0.602 (0.37–0.99)	0.731 (0.44–1.22)	0.1043
40–49	0.851 (0.55–1.32)	0.844 (0.53–1.33)	0.2326
50–59	0.751 (0.44–1.3)	0.737 (0.42–1.31)	0.4665
60~69	0.46 (0.23–0.93)	0.473 (0.22–1.03)	0.2937
≥70	0.276 (0.1–0.76)	0.344 (0.12–1.02)	0.0601
CCI (REF: CCI = 0)			
1–2	1.09 (0.69–1.72)	1.388 (0.85–2.28)	0.1939
3+	0.82 (0.36–1.86)	1.436 (0.59–3.48)	0.4229
Income (REF: ≤NTD15,840/month)			
NTD15,841–25,000	0.893 (0.6–1.34)	0.897 (0.59–1.37)	0.6141
≥NTD25,001	0.847 (0.56–1.29)	0.885 (0.57–1.38)	0.5878
Residential area (REF: 1 (most urbanized))			
2	1.031 (0.63–1.68)	0.889 (0.54–1.47)	0.6483
3	0.919 (0.47–1.79)	0.753 (0.38–1.49)	0.4147
4	1.567 (0.85–2.89)	1.639 (0.87–3.01)	0.1294
5 (least urbanized)	1.185 (0.8–1.76)	1.097 (0.73–1.66)	0.6605
ASA Scores (REF: ASA = 1)			
>1	0.723 (0.51–1.03)	0.790 (0.51–1.22)	0.2897

* All the aforementioned variables were used in multivariate analysis. REF, reference; NTD, New Taiwan Dollar; CI, confidence interval; HR, Hazard Ratio; ASA, American Society of Anesthesiologists; CCI, Charlson comorbidity index.

**Table 3:** Cox proportional hazard regression analysis of the risk of mortality among patients with nonfunctioning pituitary macroadenoma receiving different therapies.

	Crude HR (95% CI)	Adjusted HR * (95% CI)	*p*-Value
Therapeutic modality (REF: Fractionated radiotherapy)			
Stereotactic radiosurgery	0.551 (0.26–1.18)	0.36 (0.15–0.85)	0.0190
Transsphenoidal surgery	0.921 (0.63–1.34)	1.03 (0.68–1.56)	0.8948
Sex (REF: male)			
Female	0.937 (0.94–1.47)	0.903 (0.79–1.26)	0.6452
Age (REF: 1–17)			
18–29	1.65 (0.8–3.43)	0.96 (0.75–2.29)	0.2324
30–39	1.09 (0.51–2.34)	0.98 (0.45–2.15)	0.9664
40–49	2.77 (1.47–5.21)	2.03 (1.16–4.31)	0.0166
50–59	3.35 (1.70–6.16)	2.12 (1.52–6.56)	0.0020
60–69	4.34 (2.27–8.28)	2.77 (0.83–3.77)	0.0399
≥70	6.25 (3.29–8.88)	2.99 (1.41–5.38)	0.0044
CCI (REF: CCI = 0)			
1–2	3.21 (2.15–4.77)	2.08 (1.33–3.26)	0.0014
3+	6.57 (4.02–8.73)	4.56 (2.51–7.28)	<.0001
Income (REF: ≤NTD15,840/month)			
NTD15,841–25,000	0.849 (0.56–1.28)	0.953 (0.61–1.48)	0.8307
≥NTD25,001	0.649 (0.41–1.02)	0.690 (0.42–1.12)	0.1362
Regions of residence (REF: 1 (most urbanized))			
2	1.313 (0.75–2.31)	1.417 (0.79–2.54)	0.2401
3	1.804 (0.94–3.47)	1.521 (0.76–3.04)	0.2356
4	3.885 (2.18–6.92)	1.787 (0.47–5.3)	0.3018
5 (least urbanized)	1.388 (0.86–2.24)	1.08 (0.65–1.8)	0.7674
ASA Scores (REF: ASA = 1)			
>1	3.319 (2.34–4.7)	1.603 (0.99–2.6)	0.0552

* All the aforementioned variables were used in the multivariate analysis. REF, reference; NTD, New Taiwan Dollar; CI, confidence interval; HR, Hazard Ratio; ASA, American Society of Anesthesiologists; CCI, Charlson comorbidity index.

**Table 4:** Toxicities in patients with nonfunctioning pituitary macroadenoma who received fractionated radiotherapy, stereotactic radiosurgery, or transsphenoidal surgery.

	Fractionated Radiotherapy	Stereotactic Radiosurgery	Transsphenoidal Surgery	*p*-Value
	N, %	N, %	N, %	
Secondary primary brain or head and neck cancers	26 (19.55)	7 (13.21)	64 (17.68)	0.593
Hypopituitarism	14 (10.53)	5 (9.43)	37 (10.22)	0.976
Visual field deficit	47 (35.34)	12 (22.64)	95 (26.24)	0.089

## Supplementary Materials

The following are available online at https://www.mdpi.com/2077-0383/8/4/518/s1, Figure S1. Kaplan–Meier curves for all-cause mortality in patients with nonfunctioning pituitary macroadenoma who underwent stereotactic radiosurgery or transsphenoidal surgery, Figure S2. Kaplan–Meier curves for all-cause mortality in patients with nonfunctioning pituitary macroadenoma who underwent fractionated radiotherapy or transsphenoidal surgery, Figure S3 Kaplan–Meier curves for local recurrence in patients with nonfunctioning pituitary macroadenoma who underwent fractionated radiotherapy or stereotactic radiosurgery.

## Abbreviations

FRT	fractionated radiotherapy
ICD-9-CM	International Classification of Diseases, Ninth Revision, Clinical Modification
HR	hazard ratio
aHR	adjusted hazard ratio
CI	confidence interval
CCI	Charlson comorbidity index
LR	local recurrence
OS	overall survival
ASA	American Society of Anesthesiologists
Gy	gray

## References

[1] Arafah B.M., Nasrallah M.P. (2001). Pituitary tumors: Pathophysiology, clinical manifestations and management. Endocr. Relat. Cancer.

[2] Freda P.U., Wardlaw S.L. (1999). Clinical review 110: Diagnosis and treatment of pituitary tumors. J. Clin. Endocrinol. Metab..

[3] Schaller B. (2003). Gender-related differences in non-functioning pituitary adenomas. Neuro Endocrinol. Lett..

[4] Ciric I., Ragin A., Baumgartner C., Pierce D. (1997). Complications of transsphenoidal surgery: Results of a national survey, review of the literature, and personal experience. Neurosurgery.

[5] Dekkers O.M., Pereira A.M., Romijn J.A. (2008). Treatment and follow-up of clinically nonfunctioning pituitary macroadenomas. J. Clin. Endocrinol. Metab..

[6] Barker F.G., Klibanski A., Swearingen B. (2003). Transsphenoidal surgery for pituitary tumors in the United States, 1996-2000: Mortality, morbidity, and the effects of hospital and surgeon volume. J. Clin. Endocrinol. Metab..

[7] Pollock B.E., Cochran J., Natt N., Brown P.D., Erickson D., Link M.J., Garces Y.I., Foote R.L., Stafford S.L., Schomberg P.J. (2008). Gamma knife radiosurgery for patients with nonfunctioning pituitary adenomas: Results from a 15-year experience. Int. J. Radiat. Oncol. Biol. Phys..

[8] Mingione V., Yen C.P., Vance M.L., Steiner M., Sheehan J., Laws E.R., Steiner L. (2006). Gamma surgery in the treatment of nonsecretory pituitary macroadenoma. J. Neurosurg..

[9] Gupta S., Laviraj M.A., Kunhiparambath H., Sharma D., Rajendran M., Julka P.K., Rath G.K. (2017). Comparative dosimetric study of three-dimensional conformal (3DCRT), intensity modulated radiotherapy (IMRT), and volumetric modulated arc therapy (VMAT) for treatment in pituitary adenomas. J. Clin. Oncol..

[10] Mackley H.B., Reddy C.A., Lee S.Y., Harnisch G.A., Mayberg M.R., Hamrahian A.H., Suh J.H. (2007). Intensity-modulated radiotherapy for pituitary adenomas: The preliminary report of the Cleveland Clinic experience. Int. J. Radiat. Oncol. Biol. Phys..

[11] Tsang R.W., Brierley J.D., Panzarella T., Gospodarowicz M.K., Sutcliffe S.B., Simpson W.J. (1994). Radiation therapy for pituitary adenoma: Treatment outcome and prognostic factors. Int. J. Radiat. Oncol. Biol. Phys..

[12] Sheehan J.P., Niranjan A., Sheehan J.M., Jane J.A., Laws E.R., Kondziolka D., Flickinger J., Landolt A.M., Loeffler J.S., Lunsford L.D. (2005). Stereotactic radiosurgery for pituitary adenomas: An intermediate review of its safety, efficacy, and role in the neurosurgical treatment armamentarium. J. Neurosurg..

[13] Meeks S.L., Pukala J., Ramakrishna N., Willoughby T.R., Bova F.J. (2011). Radiosurgery technology development and use. J. Radiosurg. SBRT.

[14] Chiang C.J., You S.L., Chen C.J., Yang Y.W., Lo W.C., Lai M.S. (2015). Quality assessment and improvement of nationwide cancer registration system in Taiwan: A review. Jpn. J. Clin. Oncol..

[15] Wen C.P., Tsai S.P., Chung W.S. (2008). A 10-year experience with universal health insurance in Taiwan: Measuring changes in health and health disparity. Ann. Intern. Med..

[16] Shao Y.J., Chan T.S., Tsai K., Wu S.Y. (2018). Association between proton pump inhibitors and the risk of hepatocellular carcinoma. Aliment. Pharmacol. Ther..

[17] Wu S.Y., Chou H.Y., Yuh C.H., Mekuria S.L., Kao Y.C., Tsai H.C. (2018). Radiation-Sensitive Dendrimer-Based Drug Delivery System. Adv. Sci. (Weinh).

[18] Hsieh M.C., Chang W.W., Yu H.H., Lu C.Y., Chang C.L., Chow J.M., Chen S.U., Cheng Y., Wu S.Y. (2018). Adjuvant radiotherapy and chemotherapy improve survival in patients with pancreatic adenocarcinoma receiving surgery: Adjuvant chemotherapy alone is insufficient in the era of intensity modulation radiation therapy. Cancer Med..

[19] Yen Y.C., Hsu H.L., Chang J.H., Lin W.C., Chang Y.C., Chang C.L., Chow J.M., Yuan K.S., Wu A.T.H., Wu S.Y. (2018). Efficacy of thoracic radiotherapy in patients with stage IIIB-IV epidermal growth factor receptor-mutant lung adenocarcinomas who received and responded to tyrosine kinase inhibitor treatment. Radiother. Oncol..

[20] Lin Y.K., Hsieh M.C., Wang W.W., Lin Y.C., Chang W.W., Chang C.L., Cheng Y.F., Wu S.Y. (2018). Outcomes of adjuvant treatments for resectable intrahepatic cholangiocarcinoma: Chemotherapy alone, sequential chemoradiotherapy, or concurrent chemoradiotherapy. Radiother. Oncol..

[21] Chen T.M., Lin K.C., Yuan K.S., Chang C.L., Chow J.M., Wu S.Y. (2018). Treatment of advanced nasopharyngeal cancer using low- or high-dose concurrent chemoradiotherapy with intensity-modulated radiotherapy: A propensity score-matched, nationwide, population-based cohort study. Radiother. Oncol..

[22] Chang C.L., Tsai H.C., Lin W.C., Chang J.H., Hsu H.L., Chow J.M., Yuan K.S., Wu A.T.H., Wu S.Y. (2017). Dose escalation intensity-modulated radiotherapy-based concurrent chemoradiotherapy is effective for advanced-stage thoracic esophageal squamous cell carcinoma. Radiother. Oncol..

[23] Charlson M., Szatrowski T.P., Peterson J., Gold J. (1994). Validation of a combined comorbidity index. J. Clin. Epidemiol..

[24] Chen J.H., Yen Y.C., Yang H.C., Liu S.H., Yuan S.P., Wu L.L., Lee F.P., Lin K.C., Lai M.T., Wu C.C. (2016). Curative-Intent Aggressive Treatment Improves Survival in Elderly Patients With Locally Advanced Head and Neck Squamous Cell Carcinoma and High Comorbidity Index. Medicine.

[25] Fernandez A., Karavitaki N., Wass J.A. (2010). Prevalence of pituitary adenomas: A community-based, cross-sectional study in Banbury (Oxfordshire, UK). Clin. Endocrinol..

[26] Day P.F., Loto M.G., Glerean M., Picasso M.F., Lovazzano S., Giunta D.H. (2016). Incidence and prevalence of clinically relevant pituitary adenomas: Retrospective cohort study in a Health Management Organization in Buenos Aires, Argentina. Arch. Endocrinol. Metab..

[27] Onnestam L., Berinder K., Burman P., Dahlqvist P., Engstrom B.E., Wahlberg J., Nystrom H.F. (2013). National incidence and prevalence of TSH-secreting pituitary adenomas in Sweden. J. Clin. Endocrinol. Metab..

[28] Gruppetta M., Mercieca C., Vassallo J. (2013). Prevalence and incidence of pituitary adenomas: A population based study in Malta. Pituitary.

[29] Karavitaki N. (2012). Prevalence and incidence of pituitary adenomas. Ann. Endocrinol..

[30] Ntali G., Wass J.A. (2018). Epidemiology, clinical presentation and diagnosis of non-functioning pituitary adenomas. Pituitary.

[31] Penn D.L., Burke W.T., Laws E.R. (2018). Management of non-functioning pituitary adenomas: Surgery. Pituitary.

[32] Losa M., Mortini P., Barzaghi R., Ribotto P., Terreni M.R., Marzoli S.B., Pieralli S., Giovanelli M. (2008). Early results of surgery in patients with nonfunctioning pituitary adenoma and analysis of the risk of tumor recurrence. J. Neurosurg..

[33] O’Sullivan E.P., Woods C., Glynn N., Behan L.A., Crowley R., O’Kelly P., Smith D., Thompson C.J., Agha A. (2009). The natural history of surgically treated but radiotherapy-naive nonfunctioning pituitary adenomas. Clin. Endocrinol..

[34] Dekkers O.M., van der Klaauw A.A., Pereira A.M., Biermasz N.R., Honkoop P.J., Roelfsema F., Smit J.W., Romijn J.A. (2006). Quality of life is decreased after treatment for nonfunctioning pituitary macroadenoma. J. Clin. Endocrinol. Metab..

[35] Churilla T.M., Chowdhury I.H., Handorf E., Collette L., Collette S., Dong Y., Alexander B.M., Kocher M., Soffietti R., Claus E.B. (2018). Comparison of Local Control of Brain Metastases With Stereotactic Radiosurgery vs Surgical Resection: A Secondary Analysis of a Randomized Clinical Trial. JAMA Oncol..

[36] Larson D.A., Flickinger J.C., Loeffler J.S. (1993). The radiobiology of radiosurgery. Int. J. Radiat. Oncol. Biol. Phys..

[37] Hall E.J., Brenner D.J. (1993). The radiobiology of radiosurgery: Rationale for different treatment regimes for AVMs and malignancies. Int. J. Radiat. Oncol. Biol. Phys..

[38] Tome W.A. (2009). Universal survival curve and single fraction equivalent dose: Useful tools in understanding potency of ablative radiotherapy: In regard to Parks et al. (Int J Radiat Oncol Biol Phys 2008;70:847-852). Int. J. Radiat. Oncol. Biol. Phys..

[39] Park C., Papiez L., Zhang S., Story M., Timmerman R.D. (2008). Universal survival curve and single fraction equivalent dose: Useful tools in understanding potency of ablative radiotherapy. Int. J. Radiat. Oncol. Biol. Phys..

[40] Kajiwara K., Saito K., Yoshikawa K., Ideguchi M., Nomura S., Fujii M., Suzuki M. (2010). Stereotactic radiosurgery/radiotherapy for pituitary adenomas: A review of recent literature. Neurol. Med. Chir..

[41] Brada M., Rajan B., Traish D., Ashley S., Holmes-Sellors P.J., Nussey S., Uttley D. (1993). The long-term efficacy of conservative surgery and radiotherapy in the control of pituitary adenomas. Clin. Endocrinol..

[42] Chang E.F., Zada G., Kim S., Lamborn K.R., Quinones-Hinojosa A., Tyrrell J.B., Wilson C.B., Kunwar S. (2008). Long-term recurrence and mortality after surgery and adjuvant radiotherapy for nonfunctional pituitary adenomas. J. Neurosurg..

[43] Sheehan J.P., Pouratian N., Steiner L., Laws E.R., Vance M.L. (2011). Gamma Knife surgery for pituitary adenomas: Factors related to radiological and endocrine outcomes. J. Neurosurg..

[44] Liscak R., Vladyka V., Marek J., Simonova G., Vymazal J. (2007). Gamma knife radiosurgery for endocrine-inactive pituitary adenomas. Acta Neurochir. (Wien).

