# Supplementary material for: Results of Treatment with Modern Fractionated Radiotherapy, Contemporary Stereotactic Radiosurgery, and Transsphenoidal Surgery in Nonfunctioning Pituitary Macroadenoma
Source: J Clin Med. 2019 Apr 16;8(4):518. doi: 10.3390/jcm8040518 (PMC6517942; doi:10.3390/jcm8040518)
Supplement: Supplementary file 1 [file jcm-08-00518-s001.pdf]

Supplementary

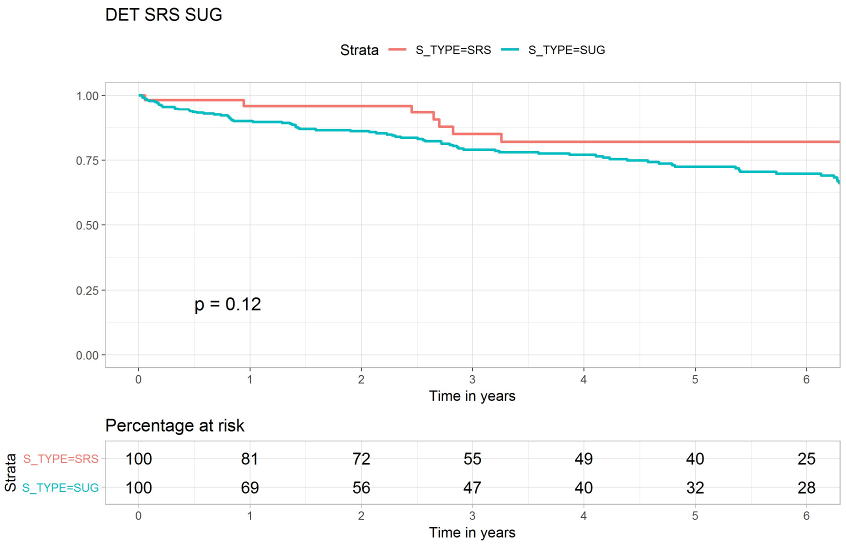

Figure S1. Kaplan–Meier curves for all-cause mortality in patients with nonfunctioning pituitary macroadenoma who underwent stereotactic radiosurgery or transsphenoidal surgery.

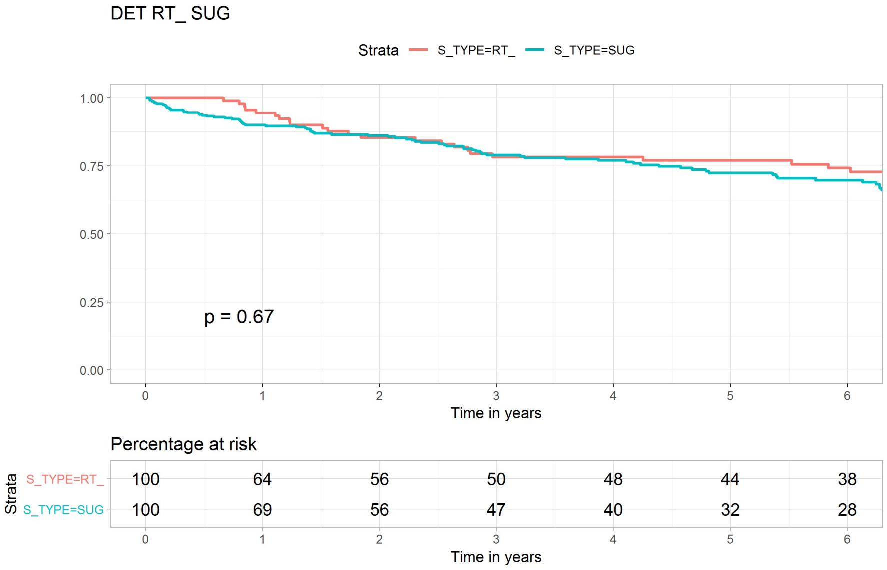

Figure S2. Kaplan–Meier curves for all-cause mortality in patients with nonfunctioning pituitary macroadenoma who underwent fractionated radiotherapy or transsphenoidal surgery.

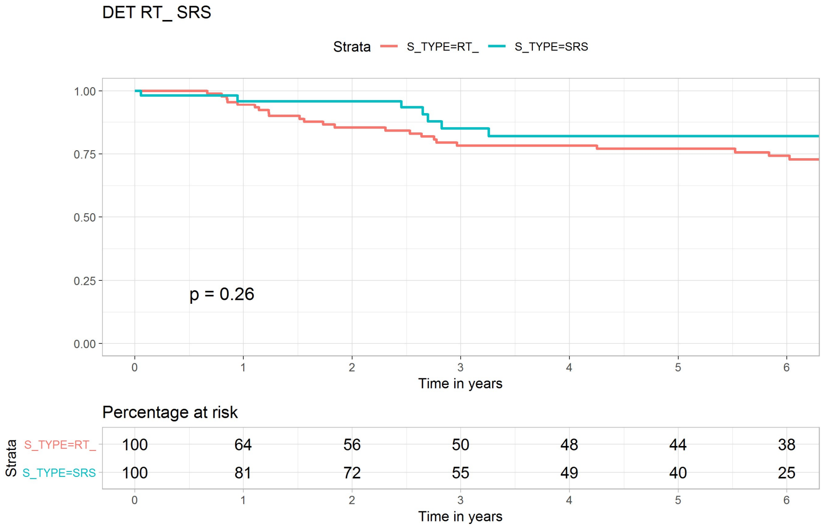

Figure S3. Kaplan–Meier curves for local recurrence in patients with nonfunctioning pituitary macroadenoma who underwent fractionated radiotherapy or stereotactic radiosurgery.
